# Effects of Dabigatran in Mouse Models of Aging and Cerebral Amyloid Angiopathy

**DOI:** 10.3389/fneur.2019.00966

**Published:** 2019-09-27

**Authors:** Neethu Michael, Mher Mahoney Grigoryan, Kelley Kilday, Rachita K. Sumbria, Vitaly Vasilevko, Joanne van Ryn, David H. Cribbs, Annlia Paganini-Hill, Mark J. Fisher

**Affiliations:** ^1^Department of Neurology, University of California, Irvine, Irvine, CA, United States; ^2^Institute for Memory Impairments and Neurological Disorders, University of California, Irvine, Irvine, CA, United States; ^3^Department of Biopharmaceutical Sciences, School of Pharmacy and Health Sciences, Keck Graduate Institute, Claremont, CA, United States; ^4^Department of Cardiometabolic Research, Boehringer Ingelheim, Hanover, Germany; ^5^Department of Pathology and Laboratory Medicine, University of California, Irvine, Irvine, CA, United States; ^6^Department of Anatomy and Neurobiology, University of California, Irvine, Irvine, CA, United States

**Keywords:** aging, cerebral amyloid angiopathy, cerebral microhemorrhage, dabigatran, direct thrombin inhibitor, intracerebral hemorrhage

## Abstract

Oral anticoagulants are a critical component of stroke prevention, but carry a risk of brain hemorrhage. These hemorrhagic complications tend to occur in elderly individuals, especially those with predisposing conditions such as cerebral amyloid angiopathy (CAA). Clinical evidence suggests that non-vitamin K antagonist oral anticoagulants are safer than traditional oral anticoagulants. We analyzed whether the anticoagulant dabigatran produces cerebral microhemorrhage (the pathological substrate of MRI-demonstrable cerebral microbleeds) or intracerebral hemorrhage in aged mice with and without hemorrhage-predisposing angiopathy. We studied aged (22 months old) Tg2576 (a model of CAA) and wild-type (WT) littermate mice. Mice received either dabigatran etexilate (DE) (Tg *N* = 7; WT *N* = 10) or vehicle (Tg *N* = 9; WT *N* = 7) by gavage for 4 weeks. Anticoagulation effects of DE were confirmed using thrombin time assay. No mice experienced intracerebral hemorrhage. Cerebral microhemorrhage analysis, performed using Prussian-blue and H&E staining, showed no significant change in either number or size of cerebral microhemorrhage in DE-treated animals. Analysis of biochemical parameters for endothelial activation (ICAM-1), blood-brain barrier disruption (IgG, claudin-5, fibrinogen), microglial activation (Iba-1), or astrocyte activation (GFAP) showed neither exacerbation nor protective effects of DE in either Tg2576 or WT mice. Our study provides histological and biochemical evidence that aged mice, with or without predisposing factors for brain hemorrhage, tolerate anticoagulation with dabigatran. The absence of dabigatran-induced intracerebral hemorrhage or increased frequency of acute microhemorrhage may provide some reassurance for its use in high-risk patient populations.

## Introduction

Dabigatran is a direct thrombin inhibitor and is indicated in patients with non-valvular atrial fibrillation for prevention of ischemic stroke (1–3). It is highly selective for thrombin, with rapid and reversible inhibition (4), and has no interaction with other enzymes involved in the coagulation cascade (5). Dabigatran inhibits tissue factor-induced thrombin generation and thrombin-induced platelet aggregation (4). Dabigatran has also shown an anti-inflammatory effect after its long-term treatment in a mouse model of Alzheimer's disease (6). It is not orally absorbed due to its polarity; dabigatran etexilate (DE), the prodrug of dabigatran, can be used orally (5, 7). This prodrug approach allows long-lasting anticoagulation (4) and has no food-drug interactions, and hence does not require routine monitoring (8).

Thrombin is a serine protease in the coagulation cascade which mediates conversion of fibrinogen to fibrin. Thrombin has been implicated in intracerebral hemorrhage (ICH) pathogenesis and mediates inflammation by activating protease-associated receptor-1 (PAR-1) (9, 10). Thrombin expression increases after ICH (11), and upregulation occurs early following ICH. The temporal pattern of thrombin expression is associated with brain edema formation (12). Via PAR signaling, thrombin also affects a wide variety of other disease entities (13), including cancer (14).

Aging and cerebral amyloid angiopathy (CAA) are two major risk factors of ICH (15–19), both of which result in brain microvessels susceptible to develop cerebral microhemorrhages (CMH). CAA is a small vessel disease characterized by deposition of β-amyloid in the cerebral vasculature (20). Spontaneous and anticoagulant-induced CMH are common in both these settings, and current research suggests a link between CMH and increased risk of ICH (21–23).

Thrombin has a pleiotropic role, and selective inhibition of thrombin may have beneficial effects in stroke prevention. Hence, we designed the current study to determine the effects of dabigatran on spontaneous CMH in aged Tg2576 transgenic mice, a model of Alzheimer's disease and CAA (24, 25), with progressive age-related accumulation of Aβ plaques (26–28). We hypothesized that given its anti-thrombin effect, dabigatran does not increase the number or size of spontaneous CMH. We used Tg2576 and wild-type (WT) littermate mice aged 22 months (comparable to humans aged 60–65 years) to mimic the scenario of elderly patients with predisposing conditions on anticoagulation therapy.

## Methods

### Animals

All experimental procedures were approved by the University of California, Irvine, Institutional Animal Care and Use Committee. To study the effect of dabigatran on spontaneous CMH development, we used an amyloid precursor protein transgenic (Tg2576) mouse model that develops CAA and spontaneous CMH and their wild type (WT) littermates. All mice used were 22 months old at the start of the experiment.

### Pretreatment With Oral Anticoagulant Dabigatran Etexilate

Dabigatran etexilate (BIBR1048MS, Boehringer Ingelheim, Ingelheim am Rhein, Germany) suspension was freshly prepared by dissolving in a vehicle solution of 0.5% hydroxyethyl cellulose solution in distilled water and using a magnetic stirrer. Four experimental groups were: (1) Tg2576 mice receiving DE (males = 3, females = 4), (2) Tg2576 mice receiving vehicle (males = 7, females = 3), (3) WT mice receiving DE (males = 5, females = 4), and (4) WT mice receiving vehicle (males = 2, females = 5). DE groups received DE doses of 45 mg/kg body weight twice daily on Monday through Friday and a single dose of 60 mg/kg via oral gavage on Saturday and Sunday, for 4 weeks. DE dosing was adapted from previously published work (29). Control mice received equal volume of vehicle solution. All the mice were weighed before starting the oral dosing and were monitored twice a week until the end of experiment.

### Determination of Diluted Thrombin Time and Plasma Concentration of Dabigatran

Plasma diluted thrombin time (dTT) and concentration of dabigatran in the plasma were determined using Hemoclot Thrombin Inhibitors (Aniara-Hyphen Biomed, West Chester, OH) and a coagulometer (Thrombostat-2, Behnk Elektronik, Norderstedt, Germany). Blood samples were obtained from a subset of mice at different time points: at baseline (*N* = 6), 0.5 h (*N* = 5), and 1.5 h (*N* = 6) after DE oral dosing, and 1.5 h (*N* = 4) after vehicle oral dosing. After anesthetizing mice with 3% isoflurane, blood samples (70 μl, 9 vol.) were collected via retro orbital sinus using plain capillary tubes (Fisher brand, Pittsburgh, PA), and were transferred to collection tubes with 3.2% sodium citrate (1 vol., prepared in distilled water). Blood was then centrifuged for 20 min at 2,000 rpm to separate plasma for later use. Plasma dTT analysis was performed following manufacturer's instructions and results were plotted on a calibration curve generated using the calibration plasma samples from the same manufacturer (Aniara-Hyphen Biomed, West Chester, OH). Corresponding dabigatran concentration for the tested plasma was interpolated directly on the calibration curve.

### Brain Preparation

Four weeks after the start of DE oral dosing, the mice were anesthetized with a lethal dose of Euthasol (150 mg/kg, i.p.), cardiac perfusion was performed using ice cold phosphate buffered saline (PBS) for 5 min and brains were harvested immediately and processed for histochemical and biochemical analysis. Right brain hemisphere was drop-fixed in 4% paraformaldehyde (PFA) for 24 h and transferred to 15% sucrose solution prepared in PBS. When the brains sank, they were transferred to 30% sucrose solution with 0.01% sodium azide until sectioning. Left hemisphere was flash frozen using dry ice and stored at −80°C for biochemical analysis.

### Detection of Cerebral Microhemorrhage

Fixed right brain hemisphere was observed for surface macrohemorrhages. The hemisphere was later sectioned into 40 μm thick coronal sections using a freezing microtome (Sliding microtome, Thermoscientific, Grand Island, NY) and the sections were collected in PBS with 0.01% sodium azide. Every 6th section was used for Prussian blue (PB) staining and ~30 sections were analyzed per brain. PB staining was performed as described earlier (25, 30). Stained sections were observed and each CMH was photographed (Olympus BX51 microscope, Infinity 2 Camera and INIFINITY ANALYZE, 6.5.0, Lumera Corporation, ON, Canada). CMH were counted at a ×20 magnification by a blinded observer as a collection of red blood cells (RBC) that appear red-orange using hematoxylin and eosin (H&E) stain (≥5 RBC) and as clear purple-blue deposits using PB, and total number and size of CMH were determined. A size cut off (50 μm^2^) was used for considering the PB-stained CMHs. Total CMH positive area was calculated as the sum of the area of each CMH and expressed as a percentage of the total area of the brain analyzed. To determine the total area analyzed, PB-stained slides were scanned using Canon MP250 scanner (Canon, Tokyo, Japan) under 600 dots per inch (DPI), and the area of each individual section was summed using NIH ImageJ software. CMH count, CMH/section (total number of CMH/number of sections), total CMH area, percentage CMH area, and average CMH size were calculated by an observer blinded to genotype and treatment group. Every 7th section (~30 sections/brain) was used for H&E staining as described previously (30), and was performed by the research service core at UCI Medical Center's Department of Pathology & Laboratory Medicine. Stained sections were observed and analyzed as described above for PB staining.

### Immunohistochemical Staining

Immunohistochemistry was performed for Iba-1 (microglial/macrophage marker), ICAM-1 (endothelial cell activation marker), IgG (blood-brain barrier (BBB) injury marker), and glial fibrillary acidic protein (GFAP, an astrocyte marker), using one 40 μm thick coronal section per mouse, 2–2.4 mm posterior to bregma. Sections were incubated in 0.5% hydrogen peroxide in 0.1 M PBS (pH 7.4) containing 0.3% Triton X-100 (PBST) for 30 min at room temperature to block endogenous peroxidase activity. After washing with PBST, sections were incubated for 30 min with PBST containing 2% bovine serum albumin to block non-specific protein binding. Sections were then incubated overnight at 4°C with a rabbit antibody against Iba-1 (1:200 dilution; Wako Chemicals USA, Richmond, VA), rabbit monoclonal antibody against ICAM-1 (1:500 dilution Abcam, Cambridge, MA); rabbit anti-mouse IgG antibody (1:200 dilution; Jackson ImmunoResearch, West Grove, PA), or rabbit antibody against GFAP (1:2,000 dilution; Abcam, Cambridge, MA). After washing with PBST, sections were incubated at room temperature for 1 h with biotinylated anti-rabbit IgG (1:500 dilution; Jackson ImmunoResearch, West Grove, PA), followed by 1 h incubation at room temperature with ABC complex, according to manufacturer instructions (Vector Laboratories, Burlingame, CA). Sections were developed with 3,3′-diaminobenzidine (Vector Laboratories, Burlingame, CA). Sixteen images per brain section were acquired randomly at ×20 magnification, and the total positive immunoreactive area (expressed as % of the total area analyzed) was quantified using NIH ImageJ software by an observer blinded to the experimental groups.

### Western Blotting

Claudin-5 and fibrinogen were quantified using Western blot. Briefly, frozen left cerebral hemispheres were pulverized, the powder was homogenized in T-PER buffer (Thermo Fisher Scientific, Waltham, MA) with protease inhibitor cocktail (Roche Applied Science, Indianapolis, IN), and soluble fraction was collected after 100,000 g centrifugation for 1 h at 4°C. Protein concentrations for Western blot analysis were determined using the Bradford protein assay, and ~50 μg of protein was resolved on SDS-PAGE 4–12% gel (Invitrogen, Carlsbad, CA). Primary antibodies for claudin-5 (tight junction protein; Abcam, Cambridge, MA) and fibrinogen (a marker of BBB permeability; US Biological, Salem, MA) were used at 1:2,000 dilution, followed by HRP-conjugated donkey anti-rabbit secondary antibody (Jackson Immuno Research, West Grove, PA). NIH ImageJ software was used to quantify Western blot band intensities. Control protein glyceraldehyde 3-phosphate dehydrogenase (GAPDH, Santa Cruz Biotechnology, Dallas, TX) was used to adjust band intensity measurements.

### Statistical Analysis

Data are presented as mean ± SEM. Kruskal-Wallis test followed by Dunn's multiple comparison tests was used for comparison of means. Two-sided *p* < 0.05 was considered statistically significant. Statistical analyses were performed using GraphPad Prism 7.

## Results

### Survival

We observed high survival rate after DE gavage administration; all mice survived except one mouse from WT DE group, which was euthanized because it demonstrated symptoms of distress. No animals developed ICH.

### Diluted Thrombin Time and Plasma Concentration of Dabigatran

**Figure 1:**
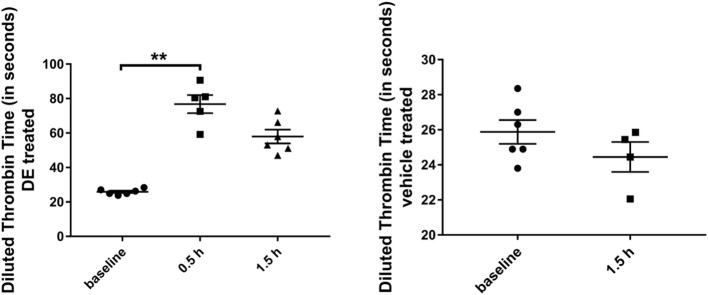
Diluted thrombin time (in seconds) in Tg2576 mice and WT littermates. Increase in diluted thrombin time at 0.5 and 1.5 h time points, compared with the baseline value in DE-treated Tg2576 mice and WT littermates. Statistical tests: Kruskal-Wallis test followed by Dunn's multiple comparison tests. Individual data points are presented with mean and SEM error bars. ^**^Indicates significant difference between means, *p* < 0.01.

In a subset of DE-treated mice analyzed for dTT, an average of 344 ± 38 ng dabigatran/ml (dTT: 76.8 ± 5.2 sec) and an average of 207 ± 29 ng dabigatran/ml (dTT: 57.9 ± 3.9 sec) were detected after 0.5 h (*N* = 5) and 1.5 h (*N* = 6) respectively; this was significantly higher compared with the baseline (*N* = 6) dTT measurements (25.9 ± 0.7 sec, Figure 1). In a subset of vehicle-treated mice analyzed, an average dTT of 24.5 ± 0.9 sec was measured 1.5 h (*N* = 4) after the gavage.

### Sub-acute Parenchymal Cerebral Microhemorrhages

**Figure 2:**
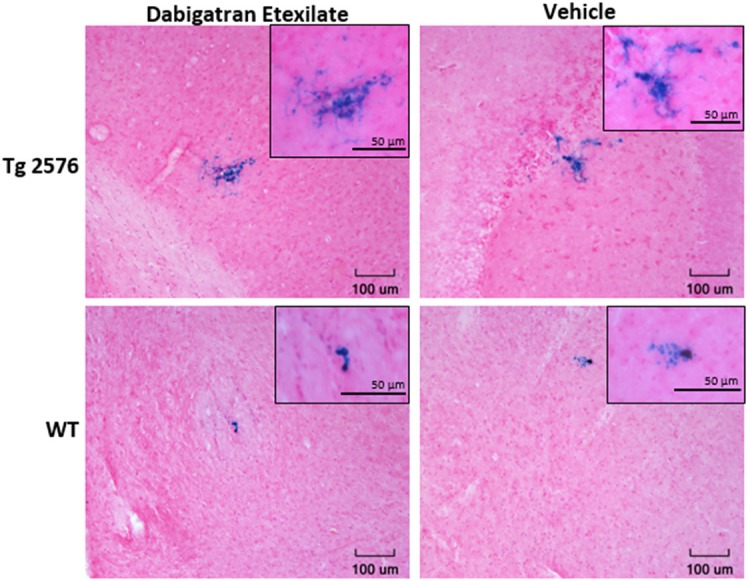
Representative images of Prussian blue-positive CMH areas from Tg2576 mice and WT littermates with or without DE treatment. Inset shows magnified (40x) image of the respective CMH.

**Figure 3:**
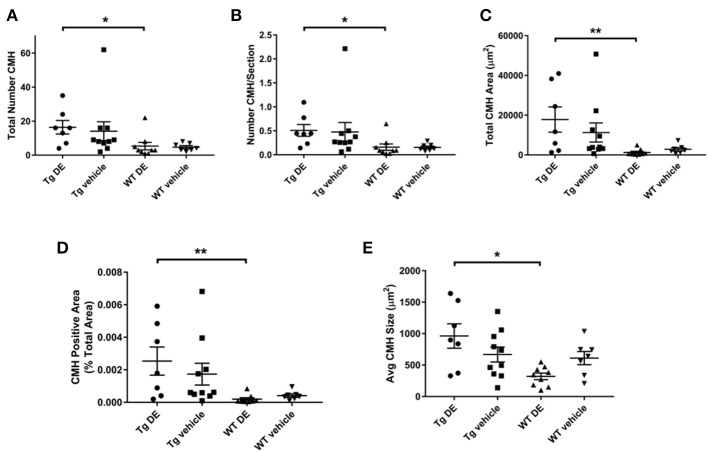
Prussian blue-positive CMH in Tg2576 and WT mice with/without DE. Significant difference in all the CMH parameters [**(A)** Total number of CMH, **(B)** Number of CMH per section, **(C)** Total CMH area, **(D)** CMH positive area (%), and **(E)** Average CMH size] of DE-treated Tg2576 mice vs. WT littermates. Statistical tests: Kruskal-Wallis test followed by Dunn's multiple comparison tests. Individual data points are presented with mean and SEM error bars. ^*^Indicates significant difference between means, *p* < 0.05. ^**^Indicates significant difference between means, *p* < 0.01.

Representative examples of PB-positive CMH from vehicle/DE-treated WT/Tg mice are shown in Figure 2. As expected, mean number of CMH (referred as mean CMH) is greater in Tg-vehicle vs. WT-vehicle mice vehicle (14.1 ± 5.5 vs. 4.7 ± 0.9, *p* = 0.3, Figure 3). DE increased mean CMH by <20% in both Tg and WT mice (16.4 ± 4.0 vs. 14.1 ± 5.5, *p* > 0.99 in Tg and 5.3 ± 2.2 vs. 4.7 ± 0.9, *p* > 0.99 in WT). A total of 115 and 141 CMH were analyzed from Tg DE (*N* = 7) and Tg vehicle (*N* = 10) groups, respectively. In Tg mice, other analyzed parameters showed no significant difference in means between the DE-treated and vehicle groups (Figure 3): CMH/section (0.51 ± 0.12 vs. 0.48 ± 0.19, *p* > 0.99), total CMH area (17,835 ± 6,351 μm^2^ vs. 11,292 ± 4,839 μm^2^, *p* > 0.99), %CMH-positive area (0.0025 ± 0.0009 vs. 0.0017 ± 0.0006, *p* > 0.99), and average CMH size (962 ± 193 μm^2^ vs. 668 ± 119 μm^2^, *p* > 0.99). In WT DE (*N* = 9) and WT vehicle (*N* = 7) groups, 48 and 33 CMH were analyzed, respectively. Similar to Tg2576 mice, WT mice showed no significant difference between the DE and vehicle groups in any of the following analyzed parameters (Figure 3): CMH/section (0.16 ± 0.064 vs. 0.16 ± 0.027, *p* > 0.99), total CMH area (1,298 ± 524 μm^2^ vs. 2,855 ± 835 μm^2^, *p* = 0.58), %CMH-positive area (0.0002 ± 0.0001 vs. 0.0004 ± 0.0001, *p* = 0.62), and average CMH size (321 ± 51 μm^2^ vs. 610 ± 105 μm^2^, *p* = 0.31). CMH were present in cortical, sub-cortical and cerebellar regions, with cortex having more CMH than other areas across all treatment groups; the exception was WT DE group, in which there was no visible difference among the three regions (One way ANOVA on total CMH present in cortex, sub-cortex and cerebellum: Tg DE: *p* = 0.002; Tg vehicle: *p* < 0.0001; WT DE: *p* > 0.8; WT vehicle: *p* = 0.009).

### Acute Parenchymal Cerebral Microhemorrhages

H&E-positive stained acute CMH were relatively few within various groups. None of the 9 WT DE mice and only one of the 7 WT vehicle mice displayed H&E-positive CMH. In Tg2576 mice, three of seven Tg DE mice and six of 10 Tg vehicle mice had H&E-positive CMH. The mean number of CMH did not differ statistically between the DE- and vehicle-treated Tg2576 mice (1.1 ± 0.8 vs. 2.2 ± 0.7, *p* > 0.9) and DE- and vehicle-treated WT littermates (0 vs. 0.2 ± 0.2, *p* > 0.9).

### Endothelial Activation, Neuroinflammation, and Blood-Brain Barrier

**Figure 4:**
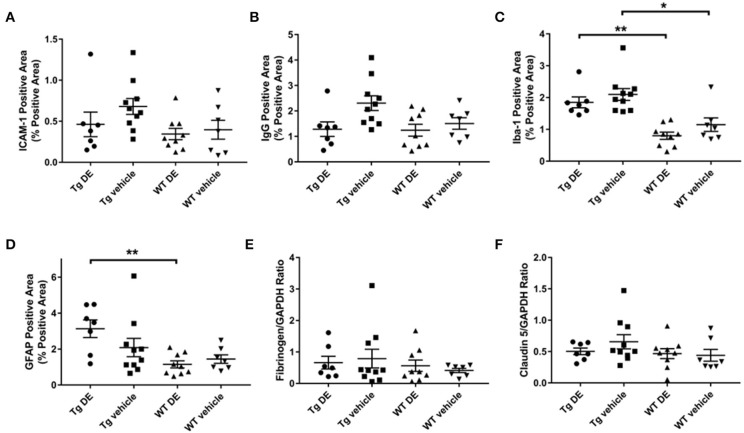
Immunohistochemical **(A–D)** and Western blot analysis **(E–F)** of endothelial activation **(A)**, blood-brain barrier structure and function **(B,E,F)**, and neuroinflammation **(C,D)** in Tg2576 and WT mice with/without DE. Significant difference in Iba-1- **(C)** and GFAP-positive **(D)** area of DE-treated Tg2576 mice and WT littermates and in Iba-1-positive area of vehicle-treated Tg2576 mice and WT littermates. Statistical tests: Kruskal-Wallis test followed by Dunn's multiple comparison tests. Individual data points are presented with mean and SEM error bars. ^*^Indicates significant difference between means, *p* < 0.05. ^**^Indicates significant difference between means, *p* < 0.01.

Immunohistochemical analysis of brains revealed no significant difference in the immunoreactivity of brain endothelial activation marker ICAM-1 between the DE- and vehicle-treated Tg2576 mice (0.5 ± 0.2% vs. 0.7 ± 0.1%) nor in their WT littermates (0.3 ± 0.1% vs. 0.4 ± 0.1%). Similarly, there was no significant difference in the total GFAP- and Iba-1-reactive areas of DE- and vehicle-treated groups of Tg (GFAP: 3.1 ± 0.5% vs. 2.0 ± 0.5%, Iba-1: 1.8 ± 0.2% vs. 2.1 ± 0.2%) and WT mice (GFAP: 1.1 ± 0.2% vs. 1.4 ± 0.2%, Iba-1: 0.8 ± 0.1% vs. 1.1 ± 0.2%). Neuroinflammatory marker Iba-1 demonstrated a significant difference between the treated and vehicle groups of Tg2576 mice and their WT littermates; astrocyte marker GFAP was significantly different between the treated groups (Figure 4). BBB structure and function assessed by immunohistochemical analysis of IgG and Western blot analysis of fibrinogen and claudin-5 showed no significant difference between DE- and vehicle-treated groups of both Tg (IgG: 1.3 ± 0.3% vs. 2.3 ± 0.3%, fibrinogen: 0.7 ± 0.2 vs. 0.8 ± 0.3, claudin-5: 0.5 ± 0.1 vs. 0.7 ± 0.1) and WT mice (IgG: 1.2 ± 0.2% vs. 1.5 ± 0.2%, fibrinogen: 0.6 ± 0.2 vs. 0.4 ± 0.1, claudin-5: 0.5 ± 0.1 vs. 0.4 ± 0.1, Figure 4).

## Discussion

The major finding of the current study is that dabigatran does not induce ICH, and neither induces nor enlarges spontaneous CMH in aged Tg2576 mice or their WT littermates. Tg2576 mice, a well-characterized model of Alzheimer's disease and CAA, progressively accumulate CMH with aging (25). In our Tg2576 mice after DE administration for 4 weeks, the mean number and size of CMH did not differ significantly between the treatment and control groups. WT mice also showed no significant effect of DE treatment on CMH formation. As expected, CMH development differed between the Tg2576 mice and WT littermates, as did the Iba-1 and GFAP immunohistochemical parameters. There was no significant difference in various markers for microglial, astrocyte and endothelial activation or BBB integrity between treated and control mice, indicating that inflammation was not induced and that BBB function remained unaltered after DE administration.

Our findings are consistent with prior published work. A cell culture-based study (31) reported a protective effect of dabigatran, inhibiting thrombin-mediated increased permeability of murine brain endothelial cells. We observed no significant increase in size of CMH in our animal models with DE treatment, suggesting a lack of increased vascular disruption with dabigatran. An MRI study (32) found that dabigatran does not promote the formation of cerebral microbleeds (the MRI signature of CMH) and does not induce ICH in APP23 mice (another mouse model of CAA) following 3–4 months of anticoagulation with DE. Our study is consistent with these findings and provides histological evidence for the MRI observations.

Our study has several limitations. PB staining, used to elucidate CMH development, characterizes CMH accumulated over the lifetime of mice, rather than simply during the 1 month treatment period. As a consequence, PB-stained microhemorrhages do not necessarily indicate lesions that developed during the course of treatment with DE. Thus, H&E stained microhemorrhages may be more meaningful in the context of this study, and it is noteworthy that there was no indication of increased number of H&E-stained lesions associated with DE treatment among either Tg or WT mice. Second, our group sizes were relatively small, and the study was thus not powered to detect some inter-group differences of potential interest. Our study lacked a positive control, i.e., we did not include use of an agent that may be more likely to have hemorrhagic consequences. Note, however, that prior work using warfarin as a positive control has reported findings similar to our own (32). In Marinescu et al., warfarin and dabigatran were administered through drinking water and chow, respectively, which enabled a longer-term treatment compared with our study, in which dabigatran was administered for a shorter period of 1 month via oral gavage. Finally, we did not attempt to distinguish primary microhemorrhages (i.e., due to an initial disruption of microvessel integrity) from secondary hemorrhages (i.e., occurring as a consequence of ischemic injury) (33), a distinction relevant in patients subject to cardiogenic stroke and hemorrhagic transformation (3).

In conclusion, we found no evidence that anticoagulation with dabigatran induces either ICH or CMH in mouse models of aging and CAA. Findings from this study and prior work may provide some reassurance for use of dabigatran in high risk populations. Further *in vivo* studies are needed to determine whether dabigatran may offer a protective effect against brain hemorrhage.

## Data Availability

The datasets generated for this study are available on request to the corresponding author.

## Ethics Statement

All experimental procedures were approved by the University of California, Irvine, Institutional Animal Care and Use Committee.

## Author Contributions

NM was responsible for study concept and design, performing *in vivo* experiments and analytic assays, statistical analysis, data analysis, and interpretation and drafting the manuscript. MG was responsible for performing *in vivo* experiments. KK and VV were responsible for performing histological and biochemical studies. RS was responsible for data interpretation and critical revision of manuscript. JR was responsible for critical revision of manuscript. DC was responsible for study concept and design, data interpretation, and critical revision of manuscript. AP-H was responsible for study concept and design, assistance with statistical analysis, data interpretation, and critical revision of manuscript. MF was responsible for study concept and design, data interpretation, study supervision and critical revision of manuscript. All authors have read and approved the final manuscript.

### Conflict of Interest Statement

MF reports grants from Boehringer-Ingelheim (48513359185) and NIH (NS20989) during the conduct of the study, and a grant from Otsuka Pharmaceutical Co. outside the submitted work. JR reports support from Boehringer-Ingelheim. DC reports a grant from NIH (NS20989) during the conduct of this study. RS reports a grant from NIH (AG055949) outside the conduct of this study. The remaining authors declare that the research was conducted in the absence of any commercial or financial relationships that could be construed as a potential conflict of interest.
